# CD8 Knockout Mice Are Protected from Challenge by Vaccination with WR201, a Live Attenuated Mutant of *Brucella melitensis*

**DOI:** 10.1155/2013/686919

**Published:** 2013-10-28

**Authors:** Samuel L. Yingst, Mina Izadjoo, David L. Hoover

**Affiliations:** ^1^Department of Bacterial Diseases, Walter Reed Army Institute of Research, Silver Spring, MD 20910-5100, USA; ^2^Armed Forces Research Institute of Medical Sciences, 315/6 Rajvithi, Bangkok 10400, Thailand; ^3^Diagnostics and Translational Research Center, Henry M. Jackson Foundation for the Advancement of Military Medicine, 401 Professional Drive, Suite 210, Gaithersburg, MD 20879, USA; ^4^DHMD Consulting, LLC, 13725 Drake Drive, Rockville, MD 20853, USA

## Abstract

CD8+ T cells have been reported to play an important role in defense against *B. abortus* infection in mouse models. In the present report, we use CD8 knockout mice to further elucidate the role of these cells in protection from *B. melitensis* infection. Mice were immunized orally by administration of *B. melitensis* WR201, a purine auxotrophic attenuated vaccine strain, then challenged intranasally with *B. melitensis* 16M. In some experiments, persistence of WR201 in the spleens of CD8 knockout mice was slightly longer than that in the spleens of normal mice. However, development of anti-LPS serum antibody, antigen-induced production of **γ**-interferon (IFN-**γ**) by immune splenic lymphocytes, protection against intranasal challenge, and recovery of nonimmunized animals from intranasal challenge were similar between normal and knockout animals. Further, primary *Brucella* infection was not exacerbated in perforin knockout and Fas-deficient mice and these animals' anti-*Brucella* immune responses were indistinguishable from those of normal mice. These results indicate that CD8+ T cells do not play an essential role as either cytotoxic cells or IFN-**γ** producers, yet they do participate in a specific immune response to immunization and challenge in this murine model of *B. melitensis* infection.

## 1. Introduction

Brucellosis causes loss of livestock productivity and threatens human health worldwide [1]. The threat is most pronounced in developing nations, but even Europe and North America remain at significant risk [2, 3]. The predominant *Brucella* species in both animal and human infections is *Brucella melitensis *[4]. A vaccine for *B. melitensis* for use in humans would be a boon to millions of agriculture workers world-wide [5] and may be an important goal for protection against bioterrorism [6].

To date, the most successful brucellosis vaccine preparations (used in livestock species) have been modified live derivatives of virulent *Brucella *[7, 8]. However, some of these vaccines are pathogenic in humans [9]. Additionally, modified live vaccines may induce abortion to livestock if administered during pregnancy or to other animals in contact with pregnant animals [10, 11]. Despite being perhaps less efficacious, subunit vaccines may have safety advantages over live attenuated candidates. Delineation of the immune mechanisms responsible for vaccine-induced protection may focus subunit vaccine development by suggesting potential immune correlates and adjuvants tailored to evoke a desired response. 

Immunization with *B. melitensis* WR201, an attenuated purine auxotroph, protects mice against intranasal challenge with virulent *B. melitensis* 16M [12]. Protection is associated with production of anti-lipopolysaccharide (LPS) antibodies and production of IFN-*γ* by antigen-stimulated immune spleen cells. The contribution of CD8+ T cells in protection has not been examined in this model.

Antibody to the O-polysaccharide of *Brucella* LPS has been firmly established as an important mediator of anti-*Brucella* effects in murine models of secondary immunity [13, 14]. However, cellular immunity also plays a key role [15, 16]. The production of IFN-*γ* is essential for protection, clearance, and survival in the face of virulent *Brucella* challenge in the mouse model [17]. IFN-*γ* is produced *in vivo* predominantly by CD4+ T cells and to a lesser extent by CD8+ T cells [16, 18, 19]. Both CD8+ and CD4+ T cells respond specifically to *B. abortus* in mice, CD8+ T cells may function as specific cytotoxic cells in brucellosis caused by *B. abortus* [16, 20, 21], and one study indicated that immune modulation could result in an effective CD8+ T-cell role in secondary immunity [22]. On the other hand, other studies seem to indicate that the role of CD8+ T cells is relatively minor in the immune response to *B. abortus* [17, 23]. One virulence mechanism of both *B. abortus* [24] and *B. melitensis* [25] may be evasion of CD8+ T cell adaptive immunity, and *B. melitensis* epitopes of CD8+ T cell IFN-*γ* production and cytotoxicity have been identified [26]. In contrast, CD8+ T cells appear to be dispensable in a model of primary *B. melitensis* infection [27]. The study reported here further elucidates the role of CD8+ T cells in brucellosis by evaluating the requirement for the cell type in secondary immunity resulting from modified live organism immunization in a mouse model of *B. melitensis*.

We found that CD8+ T cells from immunized mice specifically produce significant amounts of IFN-*γ in vitro*. However, we also found that these cells are not essential for clearance of attenuated or virulent *B. melitensis* nor for WR201-induced protection against intranasal challenge. Moreover, the key CD8+ T cell mediators of cytotoxicity (perforin and Fas) appear to play no role in elimination of *B. melitensis* in these studies. These data indicate a more limited role for CD8+ T cells in secondary immunity to *B. melitensis* than what has been suggested from previously published work using *B. abortus*. 

## 2. Materials and Methods

### 2.1. Bacteria and Bacterial Products

*B. melitensis* 16M and WR201 from our culture collection were prepared as described previously [12]. WR201 from stocks frozen in 50% glycerol was incubated overnight in *Brucella* broth in a shaker flask at 37°C. One mL aliquots of this culture were then plated on *Brucella* agar and incubated at 37°C for an additional three days. The bacterial “lawn” was then scraped from the agar surface, resuspended in 0.9% sodium chloride solution (saline), pelleted, washed twice with saline, and adjusted based on optical density to 5 × 10^11^ colony forming units (CFU)/mL in saline. In our experience this is the safest and most convenient method by which to obtain brucellae at these high concentrations. On the other hand, *B. melitensis* 16M from stocks frozen in 50% glycerol was incubated overnight in *Brucella* broth in a shaker flask at 37°C, pelleted, washed with saline, and diluted to 3.3 × 10^5^ CFU/mL—a concentration easy to obtain directly from broth. CFU concentration was verified by serial dilution and plating on *Brucella* agar. Rough *Brucella* lysate (RFBL) and *Brucella* LPS were prepared as previously described [12].

### 2.2. Immunization and Challenge of Mice

 Six-week-old C57BL/6, B6.129S2-cd8a^tm1Mak^ (CD8 knockout), C57BL/6-Pfp^tm1Sdz^ (perforin knockout), and B6.MRL-Fas^lpr^ (Fas receptor deficient mutant) mice were obtained from Jackson Laboratories (Bar Harbor, ME, USA). One pair of experiments, which used males, was conducted to evaluate kinetics of immunization and challenge in CD8 knockout compared to normal C57BL/6 mice. A subsequent pair of experiments, using females, compared the kinetics of clearance of WR201 from CD8 and perforin knockout, Fas mutant, and normal C57BL/6 mice. Animals were housed in animal biosafety level 3 facilities. Immunization and challenge procedures were performed as previously described [28]. Briefly, mice were acclimated for one week, then gavaged with 200 *μ*L 2.5% sodium bicarbonate followed by 10^11^ CFU WR201 also in 200 *μ*L. Sham-immunized mice received an equal volume (200 *μ*L) of sodium bicarbonate and saline. Seven or eight weeks following immunization, mice were either euthanized to obtain tissues for *in vitro* assays or challenged. For challenge, mice were anesthetized with 0.3 mg xylazine and 1 mg ketamine. 1 × 10^4^ CFU 16M in 30 *μ*L were then administered dropwise into the external nares with a micropipette.

### 2.3. Determination of Bacterial Infection and Immune Responses

Blood was obtained by cardiac puncture from mice euthanized by CO_2_ narcosis and allowed to clot. Serum was separated by centrifugation and sterilized by filtration through 0.2 micron filters. Anti-LPS antibody titer was determined by ELISA as previously described [12]. Organs were processed and CFU-per-organ determined by serial dilution and plating as previously described [29]. 

### 2.4. Cytokine Production

 In some experiments, production of IFN-*γ* by antigen-stimulated spleen cells from immunized or sham-immunized mice was determined as previously described [12], except for the following: total spleen cells pooled from groups of 7 mice were incubated at 5 × 10^6^/well in 24 well tissue culture plates in 2 mL RPMI-1640 tissue culture medium supplemented with 10% fetal bovine serum, 2 mM L-glutamine, 50 *μ*M 2-mercaptoethanol, and 10 *μ*g/mL gentamicin with or without 2 *μ*g/mL concanavalin A (conA) or 2 *μ*g/mL RFBL. After the entire mononuclear cell population was incubated together for 24 hrs (in order to simulate the cytokine milieu that occurs *in vivo*), nonadherent cells were collected, pelleted at 1200 rpm in a clinical centrifuge (Sorvall) for 7 minutes, and separated using the MACS separation system (Miltenyi Biotech) or resuspended in fresh medium and set aside on ice (unseparated cells). Separated CD8 and CD4+ T cells and unseparated cells were then replaced at the original concentration of mononuclear cells on the adherent spleen cells and incubation was continued for additional 48 hours in order to allow for cytokine production from individual T cell subtypes. The same cells that had been incubated with conA or RFBL were again incubated with these stimulants during this additional 48-hour period and unstimulated cells were again incubated with medium only. Culture supernatant fluids were then collected and sterilized by filtration through 0.2 micron filters. IFN-*γ* concentration was determined by ELISA as previously described [12]. 

### 2.5. Flow Cytometry

 The purity of CD4 and CD8+ T cell preparations was assessed by direct two-color immunofluorescence staining. Cells were frozen in 1% dimethyl sulfoxide in cell culture medium and stored at −80°C until the day they were stained. Cells were warmed to 4°C, concentrated by centrifugation, then resuspended in 4% methanol-free formaldehyde. Cells were incubated in formaldehyde for one hour to ensure sterility, then concentrated and washed with 0.1% bovine serum albumin in phosphate buffered saline (PBS/BSA). Cells were preincubated for 15 min at 4°C with purified rat anti-mouse CD16/CD32 (Mouse FC Block) (Pharmingen) to reduce nonspecific binding. Then cells were stained for 30 min at 4°C with CD4 FITC and CD8 PE antibodies and matched isotype IgG (Pharmingen). The staining was followed by washing with PBS/BSA in order to remove unbound antibody. Cells were resuspended in PBS/BSA prior to acquisition on the flow cytometer. 10,000 events were acquired on a FACSort (Becton Dickinson Immunocytometry Systems, San Jose, CA, USA) and analyzed using CellQuest (Becton Dickinson) software. Data on the percentage of positive cells were obtained by setting a quadrant marker for nonspecific staining.

### 2.6. Statistical Methods

 In the immunization and challenge studies, data from two separate but identical experiments were combined. The intensity of organ infection at 2 weeks after oral immunization, when most organs contained brucellae, was expressed as mean +/− SD log_10_ CFU. Statistical significance of differences in means was determined by Student's *t*-test. When the raw CFU per organ was zero, the log transformed value was assigned a value of zero, but this value was used only for graphical representation, not for statistical comparison. At all time points after immunization when some animals had cleared infection from the harvested organs, the frequency of infection in each organ was determined and the significance of differences between groups was assessed using Fisher's exact test. Additionally, CFU/spleen at that time point was analyzed descriptively. IFN-*γ* concentration was expressed as mean of triplicate or duplicate samples and analyzed descriptively.

## 3. Results

### 3.1. Clearance of WR201

 In both repetitions of the experiment that we conducted to determine clearance of immunizing strain and subsequent protection against challenge infection, WR201 persisted for 8 weeks in spleens of 2 of 5 CD8 knockout mice but was cleared from all 5 normal (C57BL6/J control) animals at this time point. Intensity of infection was less than 100 CFU in the infected spleens. Using combined data from these 2 experiments, this difference is significant (*P* = 0.044, Fisher's exact test). No other organs from either group were infected at this time point. In another pair of experiments, we immunized groups of 10 female perforin knockout, Fas mutant, CD8 knockout, and normal mice. In the first of these two experiments, all 10 mice of each group had completely cleared WR201 by eight weeks. In a repetition of the experiment, we examined the infection level at seven weeks and found that WR201 persisted at low levels in a minority of spleens of all groups. Infection levels in all mice that remained infected at this time point were less than 10 CFU/organ. There was no statistically significant difference between the groups in terms of clearance or mean log CFU per spleen. These studies showed that, unlike male mice, female mice clear WR201 between 7 and 8 weeks after immunization and that perforin and Fas play no role in its clearance. 

### 3.2. Immunization and Challenge of CD8 Knockout Mice

**Figure 1:**
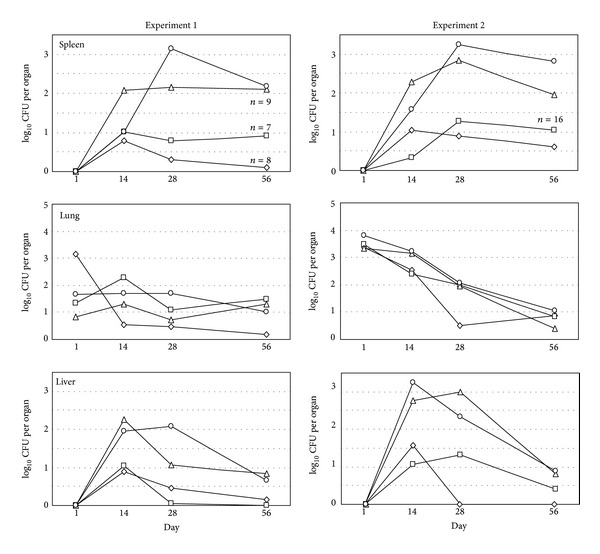
Immunization and challenge. Six-week-old male C57BL/6 and CD8 knockout mice were orally immunized with 10^11^ WR201 and eight weeks later challenged with 10^4^ *B. melitensis* 16M. Data points represent mean CFU per organ (limit of detection = 2) for 4-5 mice (first three time points) and 10 mice (56-day time point) except where noted. The figure shows naive CD8 knockout mice (circles), naive C57BL/6 mice (triangles), immune CD8 knockout mice (squares), and immune C57BL/6 mice (diamonds). In no case did mice of the same immunization status have significantly different CFU levels at a given time point in a given organ.

 We performed two separate but identical experiments in which male CD8 knockout and normal C57BL/6 mice were immunized orally with WR201 or sham-immunized. Eight weeks later, all mice were challenged intranasally with virulent 16M. We harvested spleens, lungs, and livers from mice 1 day and 2, 4 and 8 weeks following challenge to assess intensity and frequency of infection. At one day after challenge, all mice had *Brucella* in the lungs, but not in the liver or spleen (Figure 1). The course of infection in CD8 knockout and normal mice was indistinguishable (Figure 1). Immunized mice showed amelioration of spleen and liver infection, while immunization had no effect on clearance of virulent *Brucella* from the lung. In no case were culture results from C57BL/6 mice significantly different than results from CD8 knockout mice (immunized or naive) (Table 1). 

Using combined data from the individual experiments, immunization with WR201 protected both C57BL/6 and CD8 knockout mice (*P* < 0.05) from infection in the liver and spleen at 2 and 4 weeks after challenge, but CD8 knockout mice were not protected in the spleen at 2 weeks. With the data from the 2 experiments combined, there was no significant difference in protection in any organ at any time point between mice of the same immunization status, that is, naive C57BL/6 versus naive CD8 knockout mice or immunized C57BL/6 versus immunized CD8 knockout mice.

At eight weeks after challenge, using combined data from both experiments, spleens of both C57BL/6 and CD8 knockout mice were significantly protected (Table 1). There was no significant difference between mice of the same immunization status. While immunization led to significant protection of the liver of C57BL/6 mice (*P* = 0.011) but not CD8 knockout mice, the importance of this finding is uncertain, since most unimmunized animals of both strains had also cleared the infection from this organ by eight weeks.

As a further assessment of strain differences, we analyzed CFU per spleen at eight weeks after challenge for individual mice. In both immunized and nonimmunized groups, the highest splenic CFU among individual animals were found in CD8 knockout mice (not shown). Although this pattern seemed suggestive of reduced anti-*Brucella* activity in knockout mice, analysis by Mann-Whitney *U* test indicated that this trend was not significant.

### 3.3. Cellular and Humoral Immune Responses in Immunized Mice

 To determine the ability of immune T cell subpopulations to respond to *Brucella* antigens, we collected spleen cells from female C57BL/6 mice immunized 8 weeks earlier with WR201. Cells were incubated with antigen or mitogen as described in methods. In both experiments, unseparated spleen cells and CD4 and CD8+ T cells from immunized mice produced more IFN-*γ* than cells from unimmunized mice (data not shown). Cell separation was highly effective. By flow cytometric analysis, T cell subpopulations were at least 82% pure and were contaminated with less than 1% of the other T cell subset. Adherent cells alone and all cells that were incubated without RFBL failed to produce measurable IFN-*γ* (not shown). All naive and immunized cells responded similarly to conA stimulation (not shown). 

We measured anti-*Brucella *LPS IgG and IgG2a (shown, resp., in parentheses as mean calculated titer ± SEM) in CD8 knockout (7783 ± 2284, 62 ± 28, *n* = 10), perforin knockout (4421 ± 2072, 41 ± 20, *n* = 10), Fas mutant (5086 ± 1388, 67 ± 16, *n* = 7), and immunologically intact C57BL/6 (6893 ± 1055, 28 ± 5.4, *n* = 10) mice immunized 7 or 8 weeks previously with WR201. These studies did not show any significant differences in antibody levels among the groups.

## 4. Discussion

Because we found persistence of attenuated brucellae in the spleens of some male CD8 knockout mice at the time point at which others were subsequently challenged, it must be acknowledged that the apparent protection may be due in part to persistent macrophage activation. This exception may reflect a different pattern of dissemination to the spleens in CD8 knockout mice compared to the control animals. Subsequent experiments should entail a longer lag period prior to challenge in order to clarify this question. Nonetheless, in challenge experiments, naive CD8 knockout mice showed very similar kinetics of virulent brucellae dissemination and clearance as compared to normal C57BL/6 mice. It is important to note that day 1 data primarily indicates the fidelity of inoculation and has no relevance for assessment of protection.

The CD8+ T cell has been identified as an important mediator or component of the immune response to brucellosis caused by *B. abortus* in prior studies [15, 16, 20–22, 24, 25, 30, 31]. Since the species are closely related genetically, it seemed plausible that this cell type would play a similar role in the response to *B. melitensis*. If so, CD8+ T cells would seem to be a useful target for vaccine development. CD8+ T cells may be preferentially stimulated by expressing costimulatory molecules and peptides with CD8+ T cell specific epitopes in nonprofessional phagocytes (i.e., cells not differentiated as macrophages) or by delivering antigens by viral vector [32]. Preferential stimulation of CD8+ T cells could be advantageous, because stimulation of CD4+ T cells results in a mixed T helper 1 and T helper 2 response, that is, simultaneous production of IFN-*γ* and IL-10 [18, 30]. The former response is important for anti-*Brucella* immunity, while the latter may play a counterregulatory or inhibitory role.

In preliminary studies [33], we were unable to detect cytotoxicity of immune CD8+ T cells for J774 macrophages infected with rough *B. melitensis* strain WRR51. The present studies were designed to determine whether CD8+ T cells play an essential role *in vivo*, by examining WR201-induced resistance to *B. melitensis*. Our results indicate that these cells are not crucial to protection in our model. In addition to the obvious difference (*B. melitensis* versus *B. abortus*), there are a number of other differences between the methods of our studies, which showed a minimal effect, and those used by others, who observed a strong effect [15, 16, 30]. First, there may be a fundamental difference in the immune response induced by immunization with the rough strain, RB51, and a smooth strain like WR201. Second, we used mice on a C57BL/6 background, while some other studies used animals on a BALB/c background. In some systems, the BALB/c mouse shows relative persistence of infection, which may indicate inherent differences in the immune response [18]. In limited studies from our group, C57BL/6 mice tended to be more resistant to intranasal challenge than BALB/c, although differences were not significant [29]. In addition, it is possible that one study that used MHC class I knockout mice as a surrogate for CD8-deficiency may have incorrectly attributed defective *Brucella* immunity to CD8+ T cells. Although these mice are CD8+ T cell deficient, they may have other defects (e.g., poor presentation of CD1-binding antigens) that may explain their impaired ability to control infection [34]. Third, our routes of immunization and challenge are different; we immunized orally and challenged intranasally, while other groups immunized and challenged i.v. [15, 30]. It is possible that challenge by a nonmucosal route, as in these previous studies, overestimates the impact of CD8+ cells. The relative importance of variables such as these could be assessed in additional studies. Our results do not exclude a role for CD8+ T cells in naturally acquired brucellosis or in other experimental systems but indicate that they are not critical for WR201-mediated protection against intranasal *B. melitensis* challenge.

There are, however, some suggestions in these studies that CD8+ T cells may contribute to anti-*Brucella* effects in our model. In the studies in male mice, infection with WR201 was slightly, but significantly, more prolonged in CD8 knockout compared to normal animals. Similarly, there were trends toward higher intensity of infection and higher frequency of infection in spleens of both immunized and nonimmunized 16M-challenged CD8 knockout mice compared to corresponding normal mice. These differences are minor, however, and suggest a contribution of CD8+ T cells rather than a predominant effect. The near-identical infection of other organs between knockout and normal mice, however, supports the view that this contribution is of limited importance. The studies using perforin and Fas knockout mice also did not support a role for CD8+ T cell cytotoxicity as an important determinant of immunity to *Brucella*, in agreement with studies done on a *B. abortus* model [17, 35]. It is notable that we did not see differences between knockout and normal mice in our studies using female mice. While this variability may reflect inherent differences in immune responsiveness between the sexes, it may also reflect different levels of stress in these groups. Stress is experienced far more by group-housed male mice, to varying degrees by individual mice within a given group, and plays a critical role in their immune response [36]. It has been hypothesized that a key role for the CD8+ T cell is the suppression of production of T helper 2 type cytokines [30]. It may be that environmental factors, for example, stress in male mice, influence the overall trend of the T helper response and that, in certain circumstances, the CD8+ T cell plays a compensatory role.

It is possible that the role for the CD8+ T cell in murine infection with *B. abortus* is not applicable to infection with *B. melitensis* as in the present study [15, 16, 30, 31]. Perhaps the immune response to *B. abortus* relies more heavily on the CD8+ T cell. This may stem from the unusual property of *B. abortus* LPS to cross link MHC-II molecules, which could inhibit the CD4+ T cell response [37]. IFN-*γ* is essential for survival in the face of virulent *Brucella in vivo* [17] and mediates reduction in intracellular infection of cultured macrophages [38, 39]. Previous studies with CD4+ T cells, however, have shown that the CD4+ T cell is the major producer of IFN-*γ* in brucellosis [16, 18, 19]. We confirm these studies in *B. melitensis*-immunized mice and extend them by demonstrating that both CD4+ and CD8+ T cells produced IFN-*γ* in response to specific antigen, suggesting that both cell types should contribute to protective responses. These studies raise new questions about the importance of CD8+ T cells in defense against *Brucella* and suggest that the issue should be reexamined for *B. abortus*. They also suggest that a vaccine strategy aimed at sensitizing CD8+ T cells may have limited value, although this question also deserves further investigation.

## Figures and Tables

**Table 1:** Six-week-old male C57BL/6 and CD8 knockout (CD8 KO) mice were orally immunized with 10^11^ WR201 and eight weeks later challenged with 10^4^ *B.  melitensis* 16 M. Figures at 1 day and 2 weeks after challenge, when most or all animals were infected, are mean CFU ± SD. At 4 and 8 weeks, when many animals had cleared the infection and mean CFU counts became less meaningful, figures are shown as ratio of cleared/total. At day 1, *n* = 5 for C57BL/6 and *n* = 4 for CD8 KO; at 2 weeks *n* = 10 for all groups. CFU comparisons utilized Student's *t*-test while clearance comparisons utilized Fisher's exact test.

Organ	Time after challenge	C57BL/6 immunized	C57BL/6 naive	*P* value C57BL/6 immunized versus naive	CD8 KO immunized	CD8 KO naïve	*P* value CD8 KO immunized versus naive	*P* value immunized versus immunized	*P* value naïve versus naive
Liver	2 wks	1.53 ± 0.840	2.91 ± 0.646	<0.01	1.58 ± 0.887	2.88 ± 0.646	<0.05	>0.05	>0.05
	4 wks	9/10	1/10	<0.001	7/10	0/10	<0.01	>0.05	>0.05
	8 wks	17/18	11/19	<0.05	20/23	14/20	>0.05	>0.05	>0.05

Spleen	2 wks	1.29 ± 0.676	2.46 ± 1.22	<0.05	1.59 ± 0.227	1.62 ± 0.662	>0.05	>0.05	>0.05
	4 wks	6/10	1/10	<0.05	6/10	0/10	<0.01	>0.05	>0.05
	8 wks	12/18	1/19	<0.001	12/23	1/20	<0.001	>0.05	>0.05

Lung	1 day	3.38 ± 0.66	3.32 ± 0.18	>0.05	3.48 ± 0.21	3.81 ± 0.14	>0.05	>0.05	>0.05
	2 wks	2.2 ± 0.89	2.03 ± 1.17	>0.05	2.32 ± 0.82	3.08 ± 0.66	>0.05	>0.05	0.048
	4 wks	8/10	4/10	>0.05	4/8	1/10	>0.05	>0.05	>0.05
	8 wks	11/16	11/19	>0.05	10/23	10/20	>0.05	>0.05	>0.05
